# Litigation in breast surgery: unique insights from the English National Health Service experience

**DOI:** 10.1093/bjsopen/zraa068

**Published:** 2021-05-11

**Authors:** R L O’Connell, N Patani, J T Machin, T W R Briggs, T Irvine, F A MacNeill

**Affiliations:** Department of Breast Surgery, Royal Marsden NHS Foundation Trust, London, UK; Department of Breast Surgery, University College Hospital, London, UK; University College London Cancer Institute, Francis Crick Institute, London, UK; Department of Trauma and Orthopaedics, Nottingham University Hospitals NHS Trust, Nottingham, UK; National GIRFT programme, NHS England and Improvement, UK; National GIRFT programme, NHS England and Improvement, UK; Sarcoma Unit, Royal National Orthopaedic Hospital, Stanmore, UK; National GIRFT programme, NHS England and Improvement, UK; Department of Breast Surgery, Royal Surrey County Hospital, Guildford, UK; Department of Breast Surgery, Royal Marsden NHS Foundation Trust, London, UK; National GIRFT programme, NHS England and Improvement, UK

## Abstract

**Background:**

The increase in medical negligence claims against the National Health Service (NHS) over the past decade has had a detrimental impact on limited financial and human resources that could otherwise be available for direct clinical care. The aim of this study was to review litigation claims in breast surgery as part of the national Getting It Right First Time quality improvement initiative, with the aim of identifying opportunities to improve clinical practice and patient safety.

**Methods:**

All general and plastic surgical claims notified to NHS Resolution between April 2012 and April 2018 were reviewed. Claims related specifically to breast surgery were retrieved manually, and case summaries were analysed independently by two breast surgeons.

**Results:**

From 6915 claims, 449 relating to breast surgery were identified and reviewed. The mean(s.d.) claimant age was 46(13) years. The median number of claims over the 6-year period per NHS trust was 2 (range 0–22). The most frequent causes of litigation were dissatisfaction with cosmetic outcome (121 claims, 26.9 per cent) and patient-reported delays in diagnosis (121, 26.9 per cent). A large proportion of claims related to breast implant surgery (78, 17.4 per cent), and issues regarding consent/communication were common (69, 15.4 per cent). The estimated annual cost of breast surgery litigation claims ranged from £5.57 to £9.59 million (€6.35–11.02 million).

**Conclusion:**

Patient-reported delays in diagnosis and dissatisfaction with cosmetic outcome are the most common causes of litigation related to breast surgery. These key themes should be the focus for workforce learning, with the aim of improving patient care and experience.

## Teaser

Litigation due to breast surgery was analysed. There were 449 cases over 8 years. The most frequent causes of litigation were dissatisfaction with cosmetic outcome (121, 26.9 per cent) and patient-reported delay in diagnosis (121, 26.9 per cent). The estimated annual cost of breast surgery litigation claims ranged from £5.98 to £9.59 million (€6.87–11.02 million).

## Resumen

**Antecedentes:**

Durante la última década, el aumento de las reclamaciones por negligencia médica contra el Servicio Nacional de Salud (*National Health Service*, NHS) ha tenido un impacto nocivo sobre los, ya de por sí, limitados recursos humanos y financieros, que podrían haberse dedicado a la atención médica. El objetivo de este estudio fue revisar las reclamaciones judiciales en relación con la cirugía de mama con el objetivo de identificar la oportunidad de mejora en la práctica clínica y de la seguridad del paciente dentro de la iniciativa nacional *Getting It Right First Time* para la mejora de la calidad.

**Métodos:**

Se revisaron todas las reclamaciones recibidas por el NHS, tanto en servicios de cirugía general como de cirugía plástica, entre abril de 2012 y abril de 2018. Se identificaron de forma manual las reclamaciones relacionadas con la cirugía de mama y dos cirujanos de mama analizaron de forma independiente los casos.

**Resultados:**

De 6.915 reclamaciones, se identificaron y revisaron 449 relacionadas con la cirugía de mama. La edad media de los reclamantes fue de 46 años (±DE 13). La mediana de reclamaciones al NHS durante el período de seis años fue de dos (rango 0-22). Las causas más frecuentes de litigio fueron la insatisfacción con el resultado estético (*n* = 121, 26,9%) y los retrasos en el diagnóstico (*n* = 121, 26,9%). Una gran proporción de reclamaciones estaban relacionadas con el implante de prótesis (*n* = 78, 17,4%) y el proceso de consentimiento/comunicación (*n* = 69, 15,4%). El coste anual estimado de las reclamaciones relacionadas con la cirugía de mama osciló entre £5,5798-9,59 millones.

**Conclusión:**

Las causas más frecuentes de las reclamaciones realizadas por los pacientes en el ámbito de la cirugía de mama fueron los retrasos en el diagnóstico y la insatisfacción con el resultado estético. La formación del personal sanitario debería centrarse en estos temas clave con el objetivo de mejorar la atención del paciente y la experiencia.

## Introduction

Getting It Right First Time (GIRFT) is a national programme in England designed to improve the quality and efficiency of National Health Service (NHS) care by reducing unwarranted variation in clinical practice and sharing best practice between trusts. The programme aims to identify changes that will help improve patient care and outcomes, as well as deliver cost savings as a consequence of providing the best care at the first opportunity. For each of the specialties within GIRFT, a detailed data pack is compiled so that a visit can be undertaken at each hospital nationwide to allow discussion between clinicians of each unit’s performance using all national data sets^1^. Owing to the rapid rise in the volume and costs of litigation claims, clinical negligence was identified by GIRFT as a focus for quality improvement across all specialties. In addition, the increase in medicolegal claims against the NHS has a significant detrimental impact on financial and human resources that could otherwise be available for direct clinical care.

NHS Resolution (previously known as the NHS Litigation Authority) handles litigation claims on behalf of all NHS trusts in England. All trusts are obliged to inform NHS Resolution on receipt of any clinical negligence claims. The NHS Resolution claims management system provides a unique resource to understand better the causes of alleged negligence, and to identify recurring themes and opportunities to improve practice.

Over a 10-year period (2006–2007 to 2016–2017), the National Audit Office reported that the annual number of clinical negligence claims had doubled from 5300 to 10 600^2^. In 2018–2019 the annual cost of clinical claims increased to £2.4 billion (€2.8 billion; exchange rate 16 February 2021), which represents approximately 2 per cent of the entire NHS budget for that period^3^. Surgical specialties are associated with high volumes of claims^4^, with obstetrics, orthopaedics, general surgery and emergency medicine being the four highest. General surgery made up 9 per cent of cases but only 3 per cent of estimated costs, whereas obstetrics, the mostly costly specialty, accounted for 50 per cent of total litigation costs but only 10 per cent of the volume^3^.

A detailed analysis of such a database has not been performed previously on breast/general and plastic surgery to the breast. Accurate portrayal of litigation in breast surgery, regardless of whether undertaken by a breast/general or plastic surgeon, will raise awareness as to what drives patients to litigate and allow the development of strategies to improve clinical practice, increase patient satisfaction, and reduce litigation associated costs. The aim of this study was to analyse all claims related to surgery to the breast over a 6-year period to identify the causes.

## Methods

Data were retrieved for all general and plastic surgical claims notified to NHS Resolution between April 2012 and April 2018. The parameters available included claim status (whether open/ongoing or closed/resolved), incident date, notification date to NHS Resolution, and the costs incurred (including outstanding costs and those already paid in respect to damages, defence and claimant costs). The outstanding costs were estimated by NHS Resolution at the end of each financial year if the claim had not been resolved in order to estimate the annual cost. The causes of the claims were summarized in free text by the trust legal department based on the letter of claim received from the claimant. This information is then reviewed and updated by the NHS Resolution claims handlers.

Currently, NHS Resolution does not segregate breast surgery claims, which are allocated to general surgery or plastic surgery, making it challenging to identify those claims specific to breast surgery. Therefore, two breast surgeons (RLO and NP) independently undertook a systematic review of all plastic surgery and general surgery litigation cases over the six-year period and identified those cases specifically related to breast surgery using free-text summaries. The free-text field summaries associated with each claim were then analysed using a thematic analysis approach to generate a list of themes into which all claims could be classified, as well as being based on previous claims analysis in orthopaedics^5^. Owing to their multifactorial nature, several themes could be associated with any single claim. Discrepancies between the authors were discussed and consensus reached. It is noteworthy that this study did not involve reviewing complete case records from NHS Resolution or patient-level medical records from NHS trusts. All claims and costs related to the malpractice of convicted surgeon Ian Paterson were excluded from the analysis, given these were unrepresentative outliers that have been addressed separately by an independent inquiry^6^.

## Results

Between April 2012 and April 2018, 6915 claims were made: 6351 claims against general surgery and 564 claims against plastic surgery. From these, 1101 claims involved surgery to the breast. An additional 77 claims were identified as breast-related claims that were originally categorized as ‘surgical-other’ by NHS Resolution. From the 1178 claims, 729 related to Ian Paterson and were therefore excluded from downstream analysis. The remaining 449 claims comprised 244 general surgery claims (54.3 per cent of all breast claims), 128 plastic surgery claims (28.5 per cent of all breast claims), and the 77 from ‘surgical-other’ (17.1 per cent of all breast claims). Of all general surgery claims 3.5 per cent (224 of 6351) were related to breast surgery, and of all plastic surgery claims 22.7 per cent (128 of 564) were related to breast surgery. The median time between the incident occuring and claim being logged with NHS Resolution was 1027 (i.q.r. 662–1373) days. As of April 2018, 103 (22.9 per cent) of the 449 claims had been completed and closed.

### Age and sex

The mean(s.d.) age of claimants was 46(13) years; 96.9 per cent were women, with only 12 male claimants recorded.

### Trend in number and cost of claims

The annual number and cost of breast surgery-related claims over the 6 years are summarized in *Table 1*. The number of cases per year during the study period remained relatively stable, with a median of 74. The annual cost ranged from £5.57 to 9.59 million (€6.35–11.02 million).

**Table 1:** Number of litigation cases, cost, and change in litigation cost of cases between 2012 and 2018

Year	No. of claims	% change in no. of claims	Total cost (millions)		% change in total cost
			£	€	
2012–2013	80		6.62	7.61	
2013–2014	78	−3	6.80	7.81	+3
2014–2015	72	−8	5.98	6.87	−12
2015–2016	73	+1	5.57	6.40	−7
2016–2017	74	+1	6.71	7.71	+21
2017–2018	72	−3	9.59	11.02	+43
Total	449		41.27	47.42	

**Fig. 1:**
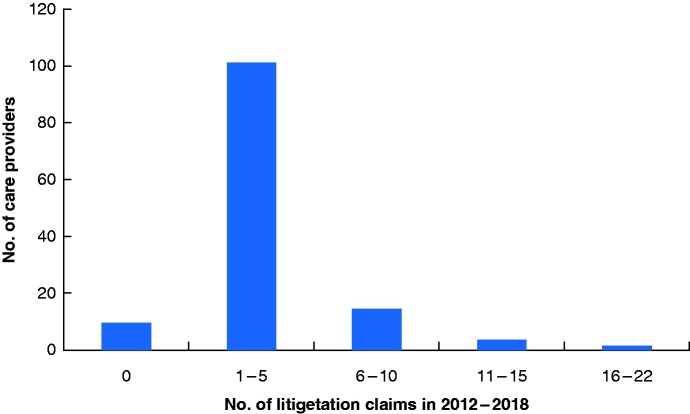
Number of litigation claims per trust between 2012 and 2018

During the data collection period there were around 130 NHS trusts providing breast surgery in England. The median number of cases per trust during the whole 6-year study interval was 2 (range 0–22). Eight trusts did not have any claims made against them and 84.1 per cent had five or fewer (*Fig. 1*). Claims were not evenly distributed between trusts: approximately half of all claims could be attributed to just 26 trusts. It is noteworthy that, for the hospital with the most claims, 12 of the 22 claims were related to Poly Implant Prothèse implants.

### Causes of litigation claims

The themes underpinning these litigation claims and their frequencies are summarized in *Table 2*. Patient-reported delays in diagnosis and dissatisfaction with cosmetic outcome were the two most common causes of litigation.

**Table 2:** Causes of 449 litigation claims related to breast surgery

Cause of litigation claim	*n*
**Delay in diagnosis or treatment**	
Delay in diagnosis	121
Delay in starting treatment	26
**Surgical decision-making or clinical judgement**	
Surgical planning decision-making	55
Clinical decision dissatisfaction	46
**Consent/communication**	
Consent	57
Communication-related issue	12
**Operative**	
Cosmetic outcome dissatisfaction	121
Incomplete excision of benign lump	10
Incomplete excision of malignant lump	12
Wrong-site surgery	4
Wrong-side surgery	0
Intraoperative injury	14
Retained foreign body	15
Breast implant-related	78
**Postoperative**	
Surgical-site infection	42*
Other infection	1
Venous thromboembolism	0
Pressure sore	4
Other complication not requiring surgery	14
Other complication requiring further surgery	27
**Miscellaneous**	
Medication error	3
Death	1
Primary breast abscess-related	5
Non-classifiable/missing information	20

Many claims involved more than one theme.

* Of the 42 infections, 19 required further surgery.

Delayed diagnosis (121 of 449 claims, 26.9 per cent) was most commonly reported by patients who presented with a symptom initially thought to be benign, but later diagnosed as malignant. Delays to treatment (26 of 449, 5.8 per cent) frequently reflected delays in starting adjuvant therapy due to postoperative complications or a delay in treating surgical-site infection.

Dissatisfaction with decision-making and clinical judgement were apparent in 22.5 per cent of claims (101 of 449). Examples of such cases included patients who felt they had the incorrect type of breast reconstruction or underwent mastectomy instead of breast conservation. Interestingly, two claims involved patients contesting whether contralateral risk-reducing mastectomy was appropriate.

Consent and communication issues were highlighted in 15.4 per cent of claims (69 of 449), and were often accompanied another complaint rather than occurring in isolation (for example inadequate consent as part of a claim relating to postoperative complications or cosmetic dissatisfaction). Strikingly, failure to consent adequately for reconstruction accounted for over half of all consent-related claims.

Claims pertaining directly to the operation itself were common, and dissatisfaction with cosmetic outcomes was noted in 121 (26.9 per cent) of all cases. These were most frequently related to breast reconstruction (66 of 121, 54.5 per cent), although many claim summaries did not specify the reconstruction type. Implants were mentioned in 78 (17.4 per cent) of all claims, of which only 10 related to breast augmentation; the vast majority were concerned with implant-based reconstruction. Breast implant-related claims cost £4.8 million (€5.5 million), indicating the high frequency and cost of litigation associated with prosthetic surgery. Of cosmetic outcome claims, 19.0 per cent (23 of 121) were related to breast reduction and 8.3 per cent (10 of 121) to augmentation surgery, compared with just 5.8 per cent (7 of 121) for mastectomy and breast-conserving surgery combined.

Although there were no cases of wrong-*side* surgery, wrong-*site* surgery (removing tissue from an incorrect site) was contested by four claimants. There were 15 claims about retained foreign objects, including wire tips, glove pieces and swabs. One claim was related to the placement of marker clips to identify the tumour cavity, which is considered best practice to facilitate adjuvant radiotherapy planning. Fourteen intraoperative injury claims included diathermy burns (8 cases), nerve compression due to improper positioning/padding (5), and corneal abrasion (1).

Surgical-site infection was the most common postoperative complication, accounting for 9.4 per cent (42 of 449) of all claims, whereas only one claim involved infection elsewhere. Other complications included haematoma, skin necrosis, flap loss, non-radiotherapy compliant expander insertion and ocular ischaemic neuropathy following postoperative hypotension. Medication errors were related to the dosing and administration of anticoagulants and antibiotics. There was one death recorded from postoperative infection after breast reduction surgery.

Regarding the 12 claims submitted by men, six were related to gynaecomastia surgery or surgery for breast/chest wall asymmetry. Two cases were related to a delay in diagnosis, one was regarding alleged inadequate management of breast cancer, and one was regarding failure to perform a contralateral prophylactic mastectomy.

### High-value cases

The 10 highest-value claims had an estimated cost of from £600 000 to £1.2 million (approximately €690 000 to €1.4 million), and are summarized in *Table 3*. Eight of these were concerned with the delayed diagnosis of primary or secondary breast cancer, indicating that diagnostic delays were not only a frequent cause of litigation but also very costly. One claim involved a lower-limb nerve palsy due to intraoperative positioning, and another was related to cosmetic dissatisfaction after breast reconstruction.

**Table 3:** The 10 highest-value cases in 2012–2018

Cause of litigation claim	Details from claim handler summary
Delay in diagnosis	Claimant alleges there was a delay in diagnosis of breast cancer leading to a delay in treatment
Delay in diagnosis	Claimant initially told presenting lump was benign; later had an excision biopsy and found to be malignant
Delay in diagnosis	Claimant alleged that there was a failure to undertake biopsy which resulted in delay in diagnosis of breast cancer
Delay in diagnosis	Claimant with *BRCA2* gene mutation due for risk reduction surgery, removed from waiting list in error; once identified, patient found to have a malignancy
Delay in diagnosis	Claimant lost to follow-up after initial breast cancer surgery; subsequent diagnosis of metastatic breast cancer
Delay in diagnosis	Delay in diagnosis of breast cancer; no further details available
Delay in diagnosis	Claimant attended symptomatic clinic and biopsy not undertaken; subsequent diagnosis of breast cancer
Cosmetic outcome dissatisfaction Consent Delay in diagnosis	Claimant alleges the consent process was inadequate, the breast reconstruction was performed poorly, and failure to diagnose capsular contracture
Intraoperative injury	Claimant developed lower-limb palsy due to incorrect fitting of calf compression garment
Cosmetic outcome Consent	Claimant alleges that the breast reconstruction she underwent was performed negligently

## Discussion

The GIRFT litigation workstream has, for the first time, provided access to all NHS England claims involving surgery on the breast over 6 consecutive years. This study has systematically reviewed claims related specifically to breast surgery, and identified recurring themes that underpin patient grievance. In breast surgery, claims were attributable most frequently to patient-reported delays in diagnosis and dissatisfaction with cosmetic outcome. These findings are consistent with other published studies that also found delayed diagnosis and poor cosmesis to be among the most frequent causes of breast surgery-related litigation in the UK^7^^,^^8^ and USA^9–11^. Inadequate consent, or failure to inform appropriately about risk, was a common theme running through many claims. This appeared particularly relevant to complex procedures and those significantly altering body image, with a disproportionate number of claims related to breast implants. Year on year, breast surgery has an estimated litigation cost in England of over £5 million (€5.7 million), and reached over £9 million (approximately €11 million) in 2017–2018. The data presented provide an important opportunity to learn from litigation, improve clinical care, increase patient satisfaction, and reduce the burden of NHS litigation-related costs.

In the UK, the evaluation of patients with breast symptoms employs the robust and sensitive protocol of triple assessment^12^. However, false-negative findings are inherent in any screening test or diagnostic process, because of either occult disease that is undiagnosable at presentation or missed diagnosis/clinical error. Despite this, lay perception is that medical tests are infallible, so any failure of diagnosis at presentation and assessment must represent negligence and so drive litigation. In a study^13^ of 2935 Australian women, 40 per cent thought screening should be 100 per cent sensitive and consequently nearly half of those surveyed believed that radiologically occult breast cancer should result in financial compensation.

Multidisciplinary team working plays an important role in mitigating clinical error and minimizing missed diagnosis. Managing patient expectations with regard to test accuracy needs to achieve a balance between understanding test limitations while maintaining confidence in accuracy. Reisch and colleagues^14^ undertook focus groups with healthcare providers regarding diagnostic errors in breast cancer. They concluded that, when communicating medical errors to patients, the complexities of breast cancer screening and diagnosis should be explained while being honest, acknowledging their feelings but focusing on the positive points going forward. Delay in diagnosis for any reason needs clear and honest explanation by senior members of the breast diagnostic team; this will help support patient understanding and may avoid litigation.

The goals of contemporary oncoplastic breast surgery are primarily to safeguard optimal local control while simultaneously minimizing cosmetic deformity. Aesthetic outcome influences psychological recovery and quality of life^15–17^; therefore, the high number of claims associated with cosmetic outcome are unsurprising. The majority of these relate to reconstructive surgery rather than simple mastectomy or breast conservation. Oncoplastic surgical techniques are wide-ranging, and tailoring the optimal approach to each individual can be complex. Detailed multidisciplinary assessment of the patient, tumour characteristics and likely adjuvant treatments (particularly radiotherapy), as well as understanding the patient’s priorities and expectations, are all essential factors. There can be significant discordance between patient and clinician assessment of aesthetic outcomes^18^, and clinicians have a duty to align patient expectations with realistic outcomes before surgery. This may involve photographs of other patients who have undergone similar surgery by the same surgeon, offering second opinions for complex cases, and multidisciplinary team discussion between breast and plastic surgeons to ensure optimal surgery is offered^19^.

This study identified a disproportionate number of claims involving breast implants. There are several potential reasons for this. First, implant-based breast reconstruction has relatively high complication rates. In the UK iBRA study^20^, which included over 2000 patients undergoing implant-based reconstruction, the rates of implant loss and hospital readmission at 3 months were 9 and 18 per cent respectively. Second, there are data to suggest that patients undergoing implant-based reconstruction have inferior satisfaction compared with those undergoing autologous reconstruction^21^, especially if radiotherapy is required^22^. This may be a consequence of failure to align patient expectations of the cosmetic outcome and adequately consent for the potential complications. Finally, the time frame of the present analysis included the Poly Implant Prothèse implants recall^23^, which led to unplanned implant (some inserted for reconstruction and others for augmentation) removals and an excess of litigation claims.

Failure to consent or warn adequately represented a recurring theme driving litigation in this study. Inadequate consent has also been identified as a common theme by others. Ford and Cooper^24^ analysed lawsuits from 11 surgical specialties in the NHS. Plastic surgery and oral and maxillofacial surgery were particularly susceptible to such claims. Providing informed consent, particularly for complex surgery such as breast reconstruction, can be time consuming and may require more than one consultation. The Royal College of Surgeons of England^25^ has published guidelines on the process of taking consent, and the General Medical Council recently updated its consent guidelines. The emphasis is for doctors to find out what matters to patients so that they can share relevant information about the benefits and harms of the proposed options and alternatives^26^. The importance of personalizing informed consent to the specific needs of the patient should now be well embedded into practice after the Montgomery ruling in 2015^27^. This stated that doctors must ensure patients are aware of any and all risks that an individual patient, not a doctor, might consider significant.

In addition, an important part of gaining informed consent is ensuring that patients understand the risk of potentially significant postoperative complications, such as infection or implant loss. If a major complication does occur, good preoperative preparation may help reduce distress and help to limit litigation.

Recommendations from the Paterson inquiry, which investigated the British surgeon Ian Paterson who was adjudged to have carried out unnecessary/inadequate breast operations, specifically recommended that patients need a period of time to reflect on their diagnosis and treatment options to ensure they are giving informed consent for treatment. Best practice should involve going through a tiered consent process, primarily undertaken in advance, for example in outpatient clinics supported by breast care nurses, and the consent process should be documented carefully in the clinic notes and patient letter. There should follow an appropriate ‘cooling-off’ period to reflect upon the information provided and to provide ample opportunity to ask questions. Consent can then be finally reconfirmed on the day of surgery.

Patients should never have to make some claims. Although there were no cases of wrong-side surgery reported to NHS Resolution during the study period, there were 15 cases of retained foreign objects and 14 intraoperative injuries. Some of these represent so-called ‘never’ events, and are best understood by root-cause analysis and ‘Swiss cheese’ error models^28^. Such significant patient safety issues can be reduced with diligent organization and good clinical practice. Use of patient safety tools and protocols such as the WHO checklist^29^ are mandatory, and some studies have demonstrated benefits^30^; however, although a breast-specific checklist has been piloted^31^, it is not in widespread use at present.

Limitations of the present study include those inherent to the database available from NHS Resolution. This repository was designed for claims management rather than a research tool for detailed causal analysis. The case summaries were entered by claims handlers rather than clinically trained staff, and were often brief. Therefore, some of the nuances of the cases may have been lost. This data set has nevertheless provided a unique opportunity to feed back individual trust-level litigation data during GIRFT visits to hospitals and to make the data available to trust medical directors. Interestingly, many of the hospital teams visited were unaware of claims against them and so had been denied the opportunity to reflect, learn, and inform clinical practice.

Further work is needed at local and national level to ensure that clinicians and management are alerted to claims in a timely manner, in order to maximize learning, improve quality of care and patient safety, and reduce litigation costs to the NHS.
